# Exploring the determinants of online purchase intention for aging-friendly products: evidence from senior consumers in urban Chinese context

**DOI:** 10.3389/fpubh.2025.1724540

**Published:** 2025-12-18

**Authors:** Liqian Yang, Ruitian Xie, Xuyun Zhao, Guanhui Zheng, Shengxiang She

**Affiliations:** 1 Guangzhou College of Technology and Business, Guangzhou, China; 2 Puning People's Hospital, Puning, China; 3 Rajapark Institute, Bangkok, Thailand

**Keywords:** aging-friendly products, digital literacy, older adults, online shopping behavior, technology anxiety, trusting beliefs in online shopping

## Abstract

This study examines the determinants of older adults’ intention to purchase aging-friendly products via online shopping platforms, focusing on senior consumers in Guangzhou, China. Drawing on the Technology Acceptance Model and the Theory of Planned Behavior, it addresses the growing need to understand digital consumption behaviors among aging populations and the factors shaping their engagement in online marketplaces. A structural model was developed to evaluate the effects of digital literacy, trusting beliefs in online shopping, technology anxiety, economic cost, and subjective norms on purchase intentions, using data collected through self-administered questionnaires from 546 adults aged 60 and above. Structural equation modeling was employed to test the hypothesized relationships. The analysis identified cognitive, emotional, social, and economic dimensions as significant contributors to intention formation, with trusting beliefs further reinforcing older adults’ willingness to engage in online purchasing. Overall, the study refines technology-acceptance frameworks for aging populations and underscores the need for digital inclusion strategies supported by public institutions, online retailers, aging-friendly manufacturers, and family caregivers.

## Introduction

The aging population is becoming increasingly serious. The World Health Organization (WHO) defines older people as those aged 60 and up (1). Many countries face significant challenges in preparing their health and social systems for the upcoming demographic shift. By the year 2030, it is projected that one in six individuals globally will be aged 60 or older. Consequently, the proportion of the population within this age group is anticipated to rise from 1 billion in 2020 to 1.4 billion. Furthermore, by 2050, the global population of individuals aged 60 and above is expected to double, reaching approximately 2.1 billion. Additionally, the number of people aged 80 and older is forecast to triple between 2020 and 2050, reaching an estimated 426 million (1). China has one of the world’s fastest-growing aging populations. Individuals over the age of 60 are predicted to account for around 28% of the overall population by 2040 (2). According to recent statistics released by the Ministry of Civil Affairs of the People’s Republic of China, the number of people aged 60 and more was 296.97 million by the end of 2023, accounting for 21.1% of the total population. There were 216.76 million people over the age of 65, accounting for 15.4% of the total population. Furthermore, the old-age dependency ratio has climbed to 22.5%, demonstrating the growing demographic pressures associated with an aging population (3). At the reginal lever, the proportion of senior residents in Guangzhou has consistently increased, from 17.27% in 2015 to 18.4% in 2019 (4). This upward trend is predicted to continue, with the proportion of people aged 60 and up increasing to 28% by 2040 (5).

The growing aging population underscores the increasing importance of aging-friendly products in enhancing the quality of life for older adults. Compared to traditional brick-and-mortar retail outlets, online stores offer significant advantages, such as lower fixed operational costs, including rent and utilities, as well as broader aging-friendly product variety and inventory availability. As a result, a substantial number of aging-oriented products have been transitioned to online platforms. Approximately 50 years ago, studies showed that a gap between older adults’ self-perceived age and their actual chronological age may influence their consumption behavior. For instance, Ying and Yao (6) found that older consumers often resisted purchasing products explicitly targeted at older adults, due to a reluctance to associate with aging. However, in recent years, increasing awareness of personal health, comfort, and convenience has gradually shifted the attitudes of older consumers. Given these evolving dynamics, the present study seeks to investigate the purchase intentions of senior citizens regarding aging-friendly products through online channels, with a particular focus on the urban aging population in Guangzhou, China.

The concept of the silver economy has been widely discussed in many fields and countries. Various scholars studied older people’s online shopping perspectives and intentions. Roy et al. (7) studied the antecedent factors that influence online purchase intention among aging consumers in India. Wu and Song (8) applied the technology acceptance model and theory of planned behavior to study the United States older adults’ online shopping continuance intention. Yap et al. (9) explored the factors that influence older adults’ intention to adopt online grocery shopping in Malaysia. In addition, Soh et al. (10) employed a unified theory of acceptance and use of technology and innovation resistance theory to discuss the senior citizens’ perception, acceptance, and willingness toward online shopping in Malaysia. Chakraborty et al. (11) compared younger adults (below 55 years old) and older adults (above 55 years old) online shopping intentions following a data breach at online retail stores in the United States. Despite the previous research studying the older people’s online purchase intention and perspectives. Most previous studies have focused on older adults’ online shopping (9), apparel shopping (12), and retail store online shopping (11). Guido et al. (13) found that the emerging generation of older adults, often perceiving themselves as younger than their chronological age or benefiting from the principles of active aging, demonstrates markedly different behavioral patterns. These individuals tend to participate in activities that earlier cohorts of older individuals would have been unlikely to consider. Moreover, aging-friendly products are often purchased not by older adults themselves, but by their adult children acting as primary decision-makers or caregivers (14, 15). In addition to cognitive and behavioral frameworks, socioemotional selectivity theory (SST) provides further insight into older adults’ online purchasing motivations. SST suggests that as individuals age, they increasingly prioritize emotionally meaningful goals and seek to avoid uncertainty or negative experiences, leading them to prefer secure, low-risk, and trust-enhancing environments when engaging with digital technologies. Recent research indicates that older adults rely more on affective cues and favor emotionally comforting decision strategies, including in consumption and technology-use contexts (16, 17). Integrating SST helps explain older consumers’ heightened emphasis on emotional security, perceived usefulness, and trustworthy online environments when evaluating aging-friendly products.

Although a growing body of research has examined older adults’ engagement with digital technologies (18, 19), existing studies remain limited in several important ways. First, prior work has seldom treated aging-friendly products as a distinct category of online consumption, despite the increasing relevance of such products for older adults’ daily living needs. Second, empirical evidence situated specifically within the Chinese context is insufficient, particularly given China’s rapid demographic aging and accelerated digitalization. Third, existing studies often focus on single dimensions such as cognitive beliefs or perceived risk, yet few have adopted an integrated framework that considers the combined influence of social, emotional, cognitive, and economic factors on older adults’ online purchasing decisions. Therefore, this study aims to address three key research gaps. First, existing literature on older adults’ online shopping behavior specifically related to aging-friendly products remains scarce. By investigating senior citizens’ online purchasing intentions toward aging-friendly products, this study seeks to contribute to this underexplored area. Second, few studies have examined the online purchasing behaviors of senior consumers within the Chinese context. This research focuses on Guangzhou, China, thereby offering localized insights and helping to bridge this geographical gap. Third, prior research has primarily concentrated on older consumers’ online purchases of general goods, such as apparel and daily necessities. Little attention has been paid to aging-friendly products specifically targeted at or purchased by older adults themselves. This study therefore provides a more nuanced understanding of older adults’ engagement with age-specific products in the digital marketplace. To present these gaps more systematically, a structured literature review table has been added to synthesize previous findings, compare theoretical and methodological approaches, and clarify how the present study extends prior work. This structure provides a clearer foundation for identifying what is known and what remains understudied in this emerging field.

## Literature review

### Aging-friendly products

Aging-friendly products refer to goods specifically designed to meet the distinct physical and psychological needs of older adults (20). These products span a wide range of categories, including food, health supplements, daily living items, medical equipment, and smart technologies tailored for senior users (21). Effective design of aging-friendly products necessitates careful consideration of the physiological characteristics of aging, addressing perceptual, functional, and emotional dimensions, as well as psychological aspects such as competence, social belonging, autonomy, and emotional wellbeing (22). To better meet the demands of older consumers, product design should emphasize several key principles: simplification of functionality, enhancement of perceptual clarity, reinforcement of safety and stability, and integration of aesthetic value (23). The primary objective of aging-friendly products is to improve the overall quality of life for older adults, promote their sense of social inclusion and subjective wellbeing, and respond to their evolving physiological and psychological needs.

### Digital literacy and technology acceptance model

Gilster (24) first introduced the concept of “digital literacy,” defining it as the ability to understand and utilize information presented in multiple formats and drawn from diverse digital sources (25). Expanding on this, Berkowsky and Czaja (26) emphasized the knowledge and competencies required to effectively navigate both hardware and software components of information and communication technologies (ICTs). For older adults, digital literacy has become increasingly essential, as numerous daily activities, such as online shopping and maintaining social connections, are now mediated through digital platforms. In this context, the perceived usefulness and perceived ease of use of digital tools ranging from hardware like smartphones and computers to software, such as e-commerce applications and social media platforms are critical factors influencing older adults’ willingness to adopt technology for purchasing aging-friendly products. Importantly, adopting online shopping does not require advanced technological expertise. Instead, a basic level of ICT competence, such as the ability to power on a smartphone, open specific applications, and connect to the internet, these ICT competences are sufficient for online shopping. Yap et al. (9) and Mohammadyari and Singh (27) found that individuals’ perceptions of a technology’s usefulness and ease of use are closely linked to their level of digital literacy. Therefore, the following hypotheses are proposed:

*H1a*: Digital literacy positively influences the perceived usefulness of older people in online purchases of aging-friendly products.

*H1b*: Digital literacy positively influences the perceived ease of use of older people in online purchases of aging-friendly products.

### Trusting beliefs in online shopping, technology acceptance model, and purchase intention

Trust refers to the expectation that the other party will act fairly and not exploit the situation for their own benefit (28). Trust is a primary predictor of the intention to behave in online shopping (29). Online shopping introduces significant privacy and security concerns. Transactions often require customers to share sensitive personal data, including names, addresses, payment information, and banking credentials. It might make them vulnerable to data breaches and misuse. As online threats continue to evolve, concerns around trust and privacy remain prominent issues (11, 30). Grimes et al. (31) found that older adults tend to be more risk-averse than younger individuals across various domains, including online behavior. Moreover, they are generally less aware of internet-related security threats (32). Trust has consistently been identified as a key determinant of user attitudes toward online activities, particularly in the context of e-commerce (33). To address these concerns, advancements in encryption and data protection technologies have enabled many e-commerce platforms to offer secure information storage and enhanced privacy protections, thereby improving user experience and increasing customer loyalty (11). The quality of online shopping services includes how easy it is to browse the website, how smooth the transaction process is, and whether the product or service delivered matches what the customer expected. When a shopping site performs well in these areas, customers are more likely to develop a stronger sense of trust in both the website and the store (34). Trust reflects the customer’s confidence that the seller will conduct transactions in line with their expectations (35). Trust in online shopping is the extent to which customers believe that the product matches what is shown on the website, serving as a sign of the seller’s honesty and indicating minimal difference between what was expected and what was received (36). Bulsara and Vaghela (37) found that a higher level of trust in online shopping increases the likelihood that customers will purchase certain products from a specific online store. Therefore, the following hypotheses are proposed:

*H2a*: Trusting beliefs in online shopping positively influences the perceived usefulness of older people in online purchases of aging-friendly products.

*H2b*: Trusting beliefs in online shopping positively influences the perceived ease of use of older people in online purchases of aging-friendly products.

*H3*: Trusting beliefs in online shopping positively influences the purchase intention of older people in online purchases of aging-friendly products.

### Technology anxiety and technology acceptance model

Technology anxiety refers to an individual’s discomfort or apprehension associated with the potential use or adoption of new technologies (38). Later, Vroman et al. (39) identified technology anxiety as a form of fear that inhibits older adults from adopting new technologies. This type of anxiety may be especially pronounced in older people who grew up in non-digital environments when they were young (40). Older people with high degrees of technology anxiety often have limited perceived skill and lower motivation to accept new technology (41). The scholars found that technology anxiety among older people is negatively related to perceived usefulness and ease of use (42, 43). Additionally, An et al. (44) found that technology anxiety among older adults negatively influence their behavior and diminish their intention to trust and engage with online services. Kim et al. (45) also found that technology-related anxiety constitutes a significant barrier to digital inclusion among older adults, particularly by undermining their trust in online shopping. An et al. (44) in their study also found that technology anxiety negatively affects both perceived usefulness and perceived ease of use in the context of digital public service adoption. Similarly, Huang (46) found that technology anxiety significantly influences perceived usefulness and ease of use, which in turn influence older adults’ intention to use smartphones. Based on the abovementioned, the following hypotheses are proposed:

*H4a*: Technology anxiety negatively influences the perceived usefulness of older people in online purchases of aging-friendly products.

*H4b*: Technology anxiety negatively influences the perceived ease of use of older people in online purchases of aging-friendly products.

### Technology acceptance model and purchase intention

Davis (47) introduced the technology acceptance model (TAM) to explain and predict users’ attitudes toward the adoption of new technologies. According to the model, two primary factors influence technology acceptance: perceived usefulness and perceived ease of use. Perceived usefulness refers to the degree to which an individual believes that using a particular technology will enhance their performance and productivity, whereas perceived ease of use pertains to the extent to which an individual believes that utilizing the technology will be free of effort. Many scholars have found that perceived usefulness and perceived ease of use positively impact the users’ attitude and intention to use particular technology. For example, Ahn et al. (48) examined Korean consumers’ intentions to use internet shopping malls and found that both perceived usefulness and perceived ease of use were positively associated with their behavioral intentions to engage with online shopping mall services. Lee (49) applied TAM to investigate the relationship between perceived usefulness and perceived ease of use toward the technology of online banking users; it found that perceived usefulness and security positively influence users’ intention to continue to use online banking service. Moreover, in the context of older people’s society, Erjavec and Manfreda (50) found that perceived usefulness of older people who are above 55 years old positively influences their online shopping intentions. Ruangkana and Kessuvan (51) in their study on the adoption behavior of older adults in Thailand, revealed that both perceived usefulness and perceived ease of use are significantly associated with senior consumers’ intentions to shop online. Additionally, Chakraborty et al. (11) investigated online shopping intentions in the context of data breaches within online retail environments and found that consumers’ trusting beliefs in shopping services are positively correlated with their intention to shop online. Another study examining the acceptance of social networking sites, a specific form of consumer technology among the post-pandemic older population in Chile, found that both perceived usefulness and perceived ease of use significantly influence older adults’ intentions to adopt and utilize such technologies (52).

Based on the abovementioned, the following hypothesis is purposed:

*H5*: Perceived usefulness positively influences the purchase intention of older people in online purchases of aging-friendly products.

*H6*: Perceived ease of use positively influences the purchase intention of older people in online purchases of aging-friendly products.

### Economic cost and purchase intention

Economic cost encompasses not only the monetary price of a product but also the financial burden perceived by consumers when initiating a purchase. It may significantly influence customers’ purchase intentions. Roy et al. (7) investigated the antecedents of online purchase intention among aging consumers in India and found that older adults are significantly more likely to engage in online shopping when they perceive financial benefits, such as cost savings and promotional discounts. According to the study by Li et al. (53), older consumers are more inclined to make online purchases when they perceive the transaction to be cost-effective. Moreover, Zhou et al. (54) investigated the willingness of older users to adopt smart home technologies and found that cost plays a critical role in shaping their adoption decisions and purchase intentions. Similarly, Kim (55) examined online shopping behaviors among individuals in their 50s and 60s in South Korea and found that economic factors, particularly price sensitivity, play a crucial role in influencing both online purchase intention and shopping satisfaction. In addition, Berg and Liljedal (56) revealed that economic aspects, such as price sensitivity and perceived value, have a significant impact on older persons’ purchase intentions, emphasizing the importance of considering economic costs when promoting aging-friendly products online. Online shops often offer more options of aging-friendly products and some items are cheaper than the offline stores. Additionally, Peng and Chen (57) found that adult children tend to purchase aging-friendly products more frequently than older adults themselves. Moreover, compared to their children, older adults exhibit greater concern regarding the costs of products and services. Therefore, the following hypothesis is proposed:

*H7*: Economic cost is positively related to the purchase intention of older people in online purchases of aging-friendly products.

### Subjective norms and purchase intention

Beck and Ajzen (58) developed the theory of planned behavior. In this theory, they defined subjective norms is perceived societal pressure to perform or not execute the activity. Later, Jacelon (59) defined that the perceived expectations of important persons influence subjective norms that an individual would behave in a specific manner, as well as perceived social pressure to engage in or abstain from a given activity. Particularly, older people’s subjective norms and behavior are influenced by their family members, friends, neighbors, or former colleagues who are important to them. Courneya et al. (60) found that compared to younger counterparts, older adults are less independent of the demands of significant others in their lives. Additionally, behavioral control is restricted by subjective norms, which are influenced by personal attitudes. Consequently, it is essential to cultivate positive behavioral processes through the cultivation of positive attitudes (53). Peña-García et al. (61) applied both the theory of planned behavior (TPB) and the technology acceptance model (TAM) in a cross-cultural study involving participants from Colombia and Spain. Their findings revealed that subjective norms significantly influenced online purchase intentions across both cultural contexts. Moreover, Bamberg et al. (62) found that subjective norms directly influence behavior, attitude, and purchase intention. However, some studies have found that subjective norms do not significantly influence purchase intentions among older adults. For instance, Wu and Song (8) examined the online shopping continuance intentions of individuals in the United States who were born before 1965. Their findings indicated that subjective norms were not significantly associated with the intention to continue online shopping. In addition, Irawan and Hurriyati (63) investigated the effects of subjective norms on online shopping intentions among consumers in Indonesia. Their study found that subjective norms did not have a significant impact on consumers’ intentions to engage in online shopping. Although previous studies have yielded mixed findings regarding the influence of subjective norms on purchase intention, the present study focuses specifically on older adults using online shopping platforms to purchase aging-friendly products. In this context, subjective norms may serve as a relevant factor influencing their purchase decisions. Moreover, older individuals who choose to shop online for aging-friendly products may experience reduced embarrassment and social pressure from significant others, as the online environment allows them to acknowledge their aging-related needs more privately and autonomously. Hence, the following hypothesis is proposed:

*H8*: Subjective norms are positively related to the purchase intention of older people in online purchases of aging-friendly products.

Table 1 lists a structured literature review.

**Table 1:** Structured literature review.

Author(s)/year	Methodology	Theory foundation	Core variables	Sample size	Country/region
Roy et al. (2023) (7)	Mixed methods: qualitative interviews and survey	Social exchange theory; online consumer behavior	Social influence, e-WOM seeking & adoption, brand loyalty, online purchase intention among ageing consumers	202 older consumers (50 + years)	India (urban ageing consumers)
Wu and Song (2021) (8)	Cross-sectional survey; SEM	Technology acceptance model; theory of planned behavior	Perceived usefulness, perceived ease of use, attitude, subjective norm, online shopping continuance intention (older adults)	366 older adults (55 + years)	United States
Yap et al. (2023) (9)	Cross-sectional questionnaire; PLS-SEM	Technology acceptance model	Perceived ease of use, perceived usefulness, functional ability, digital literacy, technology anxiety, facilitating conditions, online grocery shopping intention	302 older adults respondents	Malaysia (Klang Valley/urban context)
Ruangkana and Kessuvan (2019) (51)	Mixed methods: in-depth interviews (*n* = 30) and survey multiple regression	Technology acceptance model (TAM)	Perceived usefulness, perceived ease of use, reliability, security of payment system, consumer attitude, adoption of online purchasing	30 in-depth interviews and 150 older adults online buyers (60 + years)	Thailand (Bangkok & metropolitan)
Chakraborty et al. (2016) (11)	Online survey; PLS-SEM	Trust–risk perspective; perceived risk & severity framework	Perceived severity of data breach, perceived online shopping risk, trusting beliefs in shopping services, post-breach online shopping intention	364 respondents (159 younger <55; 205 older 55+)	United States
Xu et al. (2023) (81)	Survey of senior social media users; SEM	Flow theory; online shopping behavior	Social media usage, perceived enjoyment, flow experience, trust, purchase intention	300 social media users	Pakistan (senior social media shoppers)

## Method

### Sample and procedures

Guangzhou provides an appropriate urban context for examining aging-related online consumption in China. The city has undergone rapid demographic aging, with residents aged 60 and above accounting for 18.25% of the population as early as 2018, reflecting one of the more advanced aging profiles among major Chinese cities (64). Guangzhou is also characterized by a well-developed digital infrastructure, widespread smartphone penetration, and strong adoption of mobile-based services such as digital health platforms, online shopping, and mobile payment systems, which are deeply embedded in daily urban life (65). These features reflect broader national trends showing that older adults in China increasingly use digital technologies to support health management, communication, and daily consumption (66). At the same time, research on digital inclusion indicates that despite substantial advances in urban digitalization, older adults still face uneven levels of digital literacy, motivation, and access across regions, underscoring the importance of studying specific urban settings such as Guangzhou (67). Hence, these demographic and technological characteristics make Guangzhou a relevant and informative site for investigating older adults’ online purchasing behavior, particularly in relation to aging-friendly products. To contextualize the representativeness of the sample, the demographic structure of respondents was compared with publicly available statistics released by the Guangzhou Municipal Bureau of Statistics. The gender composition of the sample (46.3% male and 53.7% female, see Table 2) closely reflects the distribution reported in the ChinaYearbooks (68), where older adults aged 60 and above are 47.1% male and 52.9% female (69).

**Table 2:** Demographics of respondents.

Control variables	Items	Frequency	Percentage
Gender	Male	253	46.3%
	Female	293	53.7%
Age (year)	60–65	201	36.8%
	61–70	133	24.4%
	71–75	86	15.8%
	76–80	54	9.9%
	81–85	41	7.5%
	86 or above	31	5.7%
Education	Senior high school or below	236	43.2%
	Junior college	163	29.9%
	Bachelor	121	22.2%
	Master or above	26	4.8%
Individual monthly income (CNY)	Below 3,000	184	33.7%
	3,001–5,000	191	35.0%
	5,001–8,000	111	20.3%
	8,001–10,000	34	6.2%
	More than 10,001	26	4.8%
Number of children	No child	9	1.6%
	One child	164	30.0%
	Two children	255	46.7%
	Three or more children	118	21.6%
Whether living with children	No child	9	1.6%
	Living in the same city	348	63.7%
	Not living in the same city	189	34.6%

This study explores the determinants of online purchasing behavior among older adults in Guangzhou, China, focusing specifically on their purchase intentions toward aging-friendly products offered through online shopping platforms. This study targeted individuals aged 60 years and above with either official household registration or long-term residency in Guangzhou. A structured questionnaire survey was employed, utilizing both online and offline data collection methods to improve sample representativeness. Since this research involves older adults, who are considered a socially vulnerable population, the researchers consulted relevant experts prior to administering the survey and obtained ethical approval from the Institutional Review Board (IRB). The researchers also informed the participants about the objectives of the study, and obtained their informed consent before participation.

Both self-administered online responses and interviewer-assisted offline responses were collected. Data collection was concentrated in five major districts of Guangzhou: Tianhe, Yuexiu, Baiyun, Haizhu, and Panyu districts. For the online survey, the researcher initially distributed informants working as nursing staff in the senior care centers in Tianhe, Yuexiu, and Baiyun districts. They assisted the researchers to distribute the online questionnaire to their Wechat Group community. If it was necessary, assistance from adult children was allowed to support the data collection process. All online surveys were hosted on the Wenjuanxing platform, which provided secure and anonymized response tracking. In addition, convenience sampling was employed to further disseminate the survey among eligible older adults. A total of 379 valid online responses were obtained. In parallel, offline data collection was conducted at several older service community and senior care centers, located in Baiyun, Yuexiu, and Panyu districts. The researchers distributed 514 hard-copy questionnaires, yielding 167 valid responses, a response rate of 32.4%. All in-person surveys were administered under the supervision of trained research staff or volunteers. As a result, a total of 546 valid responses were obtained. This study used SPSS 27 to analyze respondents’ demographic characteristics and employed SmartPLS 4 to assess normality, reliability, validity, correlations, and path coefficients within the structural model.

The characteristics of the respondents are shown in Table 2.

### Measures

The questionnaire in this study was developed based on previously validated instruments from established research. The digital literacy scale was adapted from the work of Yap et al. (9) and comprised six items, including statements such as “I know how to activate the smartphone” and “I know how to use shopping applications (e.g., Taobao).” Technology anxiety was measured using four items, with examples including “Online shopping is somewhat scary to me” and “I feel nervous about using online shopping to buy aging-friendly products.” Perceived usefulness was assessed using five items adapted from Ruangkana and Kessuvan (51), such as “Using online shopping to buy aging-friendly products is valuable to me.” and “Using online shopping can make shopping for aging-friendly products easier.” Perceived easy of use was adapted from Roy et al. (7), Yap et al. (9), and Ruangkana and Kessuvan (51), this variable obtained four items, such as “Using online shopping would be clear and understandable when I buy aging-friendly products.” and “It is easy for me to follow the procedures when ordering aging-friendly products online.” The measurement scale of trusting beliefs in online shopping was adapted from Chakraborty et al. (11), it included four items, such as “I believe online shopping websites provide aging-friendly products as expected.” “Purchasing aging-friendly products through online channels is reliable.” Economic cost scale was adapted from the work of Li et al. (53) and obtain three items, such as “I prefer to buy aging-friendly products online if the price is right.” and “I am more willing to buy aging-friendly products online if the price is lower than the average price of products.” Subject norm consists three items, which adapted from Wu and Song (8), such as “Families support me in buying aging-friendly products online.” “People around me think buying aging-friendly products online is good.” Lastly, purchase intention obtained four items, which adapted from the work of Li et al. (53), such as “I have the intention of buying aging-friendly products through online shopping.” and “I will use online shopping to buy aging-friendly products in the near future.” Digital literacy, technology anxiety, perceived usefulness, perceived ease of use, trusting beliefs in online shopping, economic cost, subjective norms, and purchase intention toward aging-friendly products via online platforms were all measured using a five-point Likert scale, ranging from 1 (strongly disagree) to 5 (strongly agree). Given that the target respondents were Chinese-speaking older adults, the questionnaire was originally developed in English and then translated into Chinese following a standardized forward–backward translation procedure. The Chinese version was pretested with a small group of older adults to confirm item comprehension and to ensure that the wording was suitable for the senior population. After data collection, all survey items and respondent comments were translated back into English for reporting purposes.

## Results and findings

The researcher first checked whether the data followed a normal distribution, which is crucial before proceeding with further analysis. Skewness and kurtosis values were used to test this point. Skewness indicates whether the data are symmetric, while kurtosis indicates whether the data have heavy or light tails compared to a normal distribution. Values within the range of −1 to +1 indicate that the variables follow a roughly normal distribution (70). Table 3 presents the skewness and kurtosis values for all variables. The skewness values range from −0.209 to 0.89, while the kurtosis values range from −1.986 to 0.236. All univariate and multivariate skewness and kurtosis values fall within the acceptable range of −1 to +1, except for the kurtosis of the gender variable, which is slightly outside this range due to a 7.4% higher proportion of female respondents compared to male respondents. Overall, these results indicate that the data are approximately normally distributed.

**Table 3:** Normal distribution and factor loading.

Construct	Item code	Factor loading	Skewness	Kurtosis
Gender	GED	–	−0.147	−1.986
Age	AGE	–	0.886	−0.271
Education level	EDU	–	0.608	−0.735
Individual monthly income (CNY)	INCM	–	0.890	0.236
Number of children	NC	–	−0.034	−0.750
Whether living with children	WLC	–	0.334	−0.999
Digital literacy	DL1	0.768	0.189	−0.444
	DL2	0.782	0.079	−0.583
	DL3	0.76	0.018	−0.484
	DL4	0.832	0.108	−0.476
	DL5	0.77	0.048	−0.560
	DL6	0.701	0.109	−0.493
Trusting beliefs in online shopping	TBOS1	0.842	0.027	−0.339
	TBOS2	0.793	−0.013	−0.459
	TBOS3	0.822	−0.062	−0.501
	TBOS4	0.786	0.018	−0.450
Technology anxiety	TA1	0.851	0.601	−0.386
	TA2	0.823	0.508	−0.398
	TA3	0.841	0.529	−0.423
	TA4	0.783	0.629	−0.045
Perceived easy of use	PEU1	0.84	0.025	−0.492
	PEU2	0.815	0.005	−0.444
	PEU3	0.822	−0.058	−0.553
	PEU4	0.803	0.040	−0.619
Perceived usefulness	PU1	0.816	0.071	−0.494
	PU2	0.82	−0.027	−0.598
	PU3	0.803	0.041	−0.536
	PU4	0.827	0.140	−0.472
	PU5	0.791	0.090	−0.501
Economic cost	EC1	0.757	−0.070	−0.530
	EC2	0.859	0.015	−0.523
	EC3	0.806	−0.045	−0.318
Subjective norms	SN1	0.823	−0.292	−0.384
	SN2	0.837	−0.271	−0.375
	SN3	0.844	−0.211	−0.585
Purchase intention	PI1	0.83	−0.139	−0.592
	PI2	0.846	−0.160	−0.427
	PI3	0.787	−0.192	−0.545
	PI4	0.797	−0.172	−0.519

In the next step, the reliability of the sample was assessed. Cronbach’s alpha and composite reliability coefficients are widely used indicators to evaluate internal consistency. According to Cheung et al. (71), a Cronbach’s alpha value above 0.7 indicates acceptable reliability. Similarly, Hair et al. (72) suggest that composite reliability coefficients should also exceed 0.7 to confirm measurement consistency. As shown in Table 4, the Cronbach’s alpha values range from 0.737 to 0.862, the composite reliability (rho_a) values range from 0.760 to 0.871, and the composite reliability (rho_C) values range from 0.849 to 0.906. These results indicate that the reliability of the measurements in this study meets the recommended standards.

**Table 4:** Correlations among variables; reliability; and VIF values.

Constructs	GED	AGE	EDU	INCM	NC	WLC	DL	TBOS	TA	PEU	PU	EC	SN	PI
GED	1													
AGE	0.003	1												
EDU	−0.025	−0.041	1											
INCM	−0.105*	0.032	0.204^**^	1										
NC	0.050	0.015	0.042	−0.005	1									
WLC	0.083	0.088*	0.031	0.013	0.121^**^	1								
DL	−0.070	−0.025	0.029	−0.001	−0.011	−0.011	1							
TBOS	0.017	−0.053	−0.039	0.035	0.036	−0.022	0.028	1						
TA	0.021	0.014	−0.054	−0.073	0.055	−0.011	0.035	−0.017	1					
PEU	−0.022	−0.031	0.025	−0.003	0.012	−0.008	0.493^**^	0.446^**^	−0.441^**^	1				
PU	−0.030	−0.056	0.034	0.100^*^	−0.026	−0.023	0.412^**^	0.571^**^	−0.487^**^	0.521^**^	1			
EC	−0.082	0.020	0.013	0.046	0.098^*^	0.041	−0.003	−0.074	0.044	−0.041	−0.054	1		
SN	−0.027	−0.051	0.043	−0.016	−0.019	−0.003	0.070	−0.026	−0.036	0.055	0.045	0.072	1	
PI	0.006	−0.014	−0.047	0.063	0.010	−0.033	−0.040	0.300^**^	−0.34^**^	0.221^**^	0.326^**^	0.197^**^	0.247^**^	1
Cronbach’s *α*	n/a	n/a	n/a	n/a	n/a	n/a	0.862	0.826	0.843	0.838	0.807	0.737	0.783	0.832
AVE	n/a	n/a	n/a	n/a	n/a	n/a	0.593	0.658	0.681	0.672	0.658	0.654	0.697	0.665
SQ AVE	n/a	n/a	n/a	n/a	n/a	n/a	0.770	0.811	0.825	0.820	0.811	0.809	0.835	0.815
CR (rho_a)	n/a	n/a	n/a	n/a	n/a	n/a	0.870	0.829	0.847	0.839	0.871	0.760	0.786	0.839
CR (rho_c)	n/a	n/a	n/a	n/a	n/a	n/a	0.897	0.885	0.895	0.891	0.906	0.849	0.874	0.888
VIF	n/a	n/a	n/a	n/a	n/a	n/a	1.002	1.572	1.002	1.445	1.721	1.010	1.013	1.899

**p < 0.01, *p < 0.05. GED, gender; EDU, education level; INCM, individual monthly income (CNY); NC, number of child; WLC, whether living with children; DL, digital literacy; TBOS, trusting beliefs in online shopping; TA, technology anxiety; PEU, perceived ease of use; PU, perceived usefulness; EC, economic cost, SN, subjective norms; PI, purchase intentions; AVE, average variance extracted; SQ AVE, square root of AVE; CR, composite reliability; VIF, variance inflation factor.

Subsequently, the convergent validity and discriminate validity were tested. Hair et al. (70) recommend that the average variance extracted (AVE) for constructs with multiple indicators should exceed 0.5 to establish satisfactory convergent validity. As shown in Table 4, the AVE values for each construct range from 0.593 to 0.697, and the square roots of the AVE values range from 0.770 to 0.835. Moreover, factor loading is a key indicator used to evaluate convergent validity. According to Cheung et al. (71), a factor loading greater than 0.7 is ideal. Table 3 shows that all factor loadings fall between 0.701 and 0.859, thus exceeding the recommended threshold. These results confirm that the constructs in this study meet the criteria for convergent validity.

Heterotrait–Monotrait ratio (HTMT) and Fornell–Larcker are two main methods to examine the discriminant validity. According to Henseler et al. (73), it is recommended that the value of HTMT less than 0.85 indicates that the discriminant validity among constructs is good. Table 5 shows that all HTMT values of each construct are lower than the suggested threshold. Additionally, Schober et al. (74) suggested that the square root of AVE for each latent variable should be greater than the squared correlations for the other latent variables. The detailed values of the Fornell-Larcker criterion are displayed in Table 6. It is confirmed that the discriminant validity of this study meets the requirement.

**Table 5:** Heterotrait–Monotrait ratio (HTMT) matrix.

Constructs	DL	TBOS	TA	PEU	PU	EC	SN	PI
DL								
TBOS	0.08							
TA	0.061	0.04						
PEU	0.58	0.537	0.525					
PU	0.476	0.674	0.568	0.611				
EC	0.048	0.095	0.058	0.07	0.072			
SN	0.089	0.073	0.052	0.07	0.063	0.094		
PI	0.053	0.362	0.405	0.265	0.384	0.252	0.306	

**Table 6:** Fornell–Larcker criterion.

Constructs	DL	TBOS	TA	PEU	PU	EC	SN	PI
DL	0.77							
TBOS	0.032	0.811						
TA	0.033	−0.02	0.825					
PEU	0.498	0.448	−0.441	0.82				
PU	0.415	0.574	−0.488	0.523	0.811			
EC	0	−0.073	0.044	−0.037	−0.049	0.808		
SN	0.068	−0.026	−0.04	0.056	0.045	0.067	0.835	
PI	−0.038	0.302	−0.34	0.224	0.328	0.201	0.251	0.815

DL, digital literacy; TBOS, trusting beliefs in online shopping; TA, technology anxiety; PEU, perceived ease of use; PU, perceived usefulness; EC, economic cost; SN, subjective norms; PI, purchase intentions.

The variance inflation factor (VIF) is commonly used to assess multicollinearity among variables. A VIF value above 10 indicates significant collinearity issues (75). While Kock and Lynn (76) suggest that values below 3.3 are preferable. As shown in Table 4, the lowest VIF value is for technology anxiety (1.002), and the highest is for purchase intention (1.899). Since all VIF values fall below the recommended thresholds, it can be concluded that multicollinearity is not a serious concern in this study.

After confirming that the data met the requirements for normality, reliability, validity, and absence of multicollinearity, the next step involved examining the path coefficients within the structural equation model (SEM). Hypotheses 1a and 1b proposed that digital literacy has a positive effect on perceived usefulness and perceived ease of use, respectively. The results revealed significant positive relationships (*β* = 0.413; *β* = 0.499), both of which were statistically supported (*p* < 0.001). Therefore, hypotheses 1a and 1b were supported. Hypotheses 2a and 2b proposed that trusting beliefs in online shopping positively influence perceived usefulness and perceived ease of use. The results indicated significant and positive associations for both relationships (*β* = 0.551 for perceived usefulness; *β* = 0.423 for perceived ease of use), with statistical significance at the *p* < 0.001 level. Therefore, hypotheses 2a and 2b were supported. Additionally, Hypothesis 3 proposed that trusting beliefs in online shopping are positively associated with the purchase intention of aging-friendly products through online platforms. The results revealed a significant positive relationship between the two variables (*β* = 0.318, *p* < 0.001), thereby providing support for Hypothesis 3. Hypotheses 4a and 4b suggested that technology anxiety would negatively impact perceived usefulness and perceived ease of use. The findings demonstrated statistically significant negative relationships (*β* = −0.491 and *β* = −0.449, *p* < 0.001), supporting both hypotheses. These results indicate that higher levels of technology anxiety are associated with lower perceptions of usefulness and ease of use. Hypothesis 5 proposes that perceived usefulness positively influences purchase intention. The results displayed that the two variables have a positive relationship, which is also statistically significant (*β* = 0.201, *p* < 0.001). Hence, hypothesis 5 was accepted. Hypothesis 6 predicted that perceived ease of use is positively associated the purchase intention. The result indicated that there was a positive relationship between these variables (*β* = 0.026). However, the *p*-value is equal to 0.553; it’s not statistically significantly supported. Therefore, we can conclude that hypothesis 6 was not supported. Hypothesis 7 predicted that economic cost is positively associated with the purchase intention of aging-friendly products via online platforms. The results indicated a positive relationship between the variables; meanwhile, it was statistically significantly supported (*β* = 0.211, *p* < 0.001). Lastly, hypothesis 8 proposed that subjective norms positively influence purchase intention. The results showed a positive relation with *β* = 0.231, and the *p*-value also indicated a statistically significant result. Therefore, hypothesis 8 is established.

**Figure 1:**
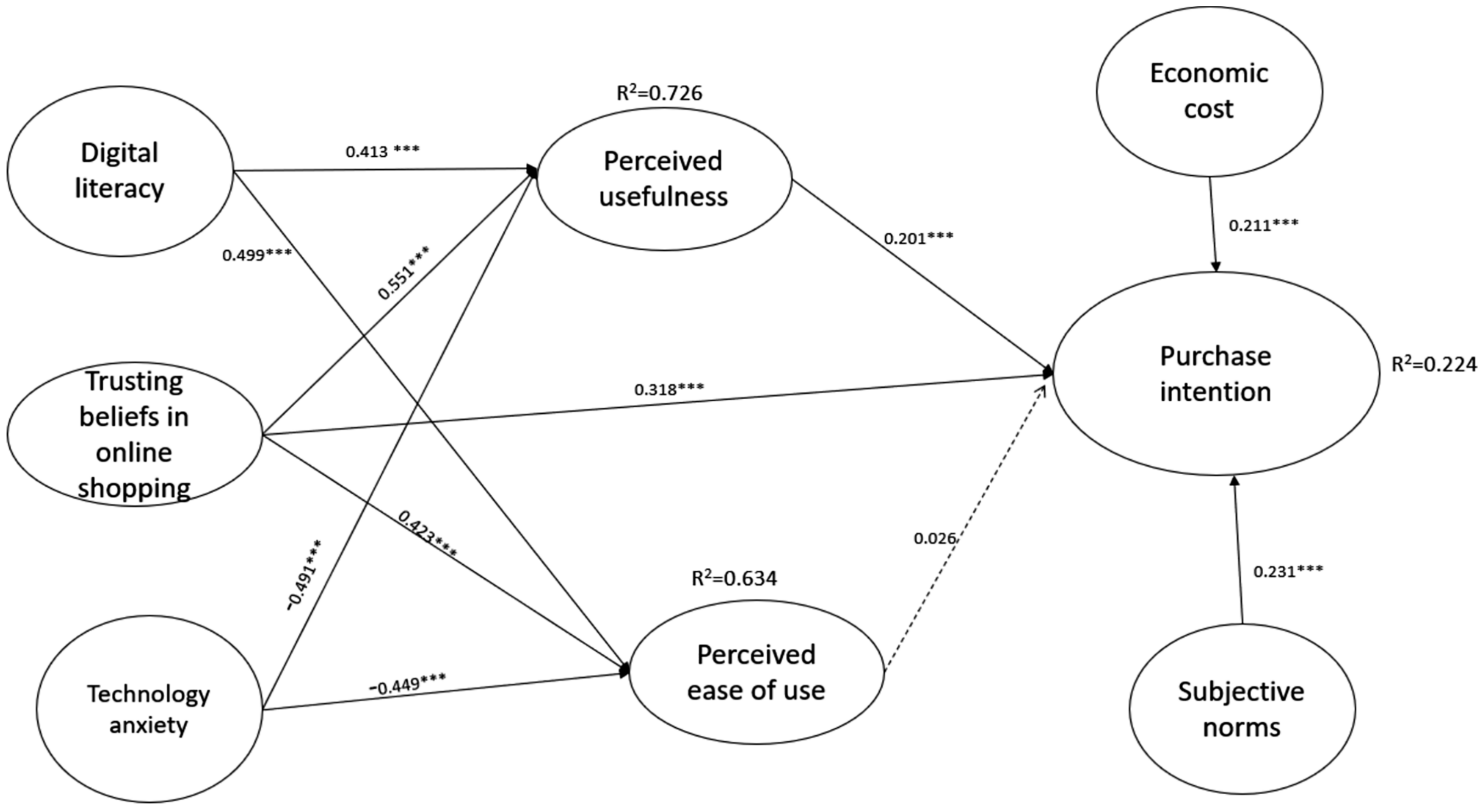
Results of the structural equation model. ***p < 0.001, solid line = significant supported; dotted line = not supported.

Moreover, the adjusted *R*^2^ of perceived usefulness is equal to 0.726, whereas the adjusted *R*^2^ of perceived ease of use is equal to 0.634, which means that digital literacy, trusting beliefs in online shopping, and technology anxiety can explain perceived usefulness by 72.6% and can explain and predict perceived ease of use by 63.4%, respectively. The adjusted *R*^2^ of purchasing intention is equal to 0.224. This means that purchase usefulness, perceived ease of use, trusting beliefs in online shopping, economic cost, and subjective norms can predict and explain purchase intention by 22.4%. The other 77.6% might be influenced by other factors. All the results information is displayed in Figure 1.

The goodness-of-fit (GoF) and the standardized root mean square residual (SRMR) are used to evaluate whether the SEM is a good fit or not. According to Henseler et al. (77), an SRMR lower than 0.08 indicates a good fit. The SRMR of this research is equal to 0.045, which is less than the threshold. Moreover, the GoF index is used to assess the fit of a model. A GoF value below 0.10 indicates poor fit, values between 0.10 and 0.25 suggest a small fit, values from 0.25 to 0.36 indicate a moderate fit, and values exceeding 0.36 reflect a strong model fit (78). The value of GoF in this study is equal to 0.386, which is a strong model fit. Based on the abovementioned condition, the research model demonstrated a good model fit.

## Discussion

Based on TAM theory, this study investigated how digital literacy, trusting beliefs in online shopping, technology anxiety, economic cost, and subjective norms drive the purchase intention among senior citizens to buy aging-friendly products through online platforms. According to findings, it was found that digital literacy, trusting beliefs in online shopping, and anxiety are antecedents of both perceived usefulness and perceived ease of use. When older adults possess enough digital literacy, they tend to be more open to adopting new technologies in the context of online shopping. Likewise, a higher level of trust in online shopping positively influences their acceptance of technology, particularly in terms of perceived usefulness and perceived ease of use. Conversely, higher levels of technology anxiety among older adults are associated with lower acceptance of online shopping technologies. Yap et al. (9) and Chakraborty et al. (11) found similar results that are consistent with this study. In the present study, it was also found that trusting beliefs in online shopping have a direct positive effect on purchase intention among older adults. Similarly, Zhang et al. (79) reported that in the context of metaverse shopping, customers’ purchase intentions are positively influenced by perceived trustworthiness. These findings provide further empirical support for the role of trust in shaping consumer behavior across different digital environments.

This study found that although perceived ease of use had a positive effect on older adults’ intention to purchase aging-friendly products online, the relationship was not statistically significant. This indicates that, compared to other influencing factors, perceived ease of use did not play a critical role in shaping purchase intention in this context. The results are consistent with the study of Wang et al. (80), who found that the influence of perceived ease of use on older adults’ willingness to continue using wearable devices was not significant within the context of senior care institutions. Li et al. (81) also found that perceived ease of use does not significantly influence older adults to use aging health care products. However, this study found that perceived usefulness has a significant positive correlation with the older adults’ intentions to purchase aging-friendly products through online shopping. Xu et al. (82) also found consistent results. Enhancing perceived usefulness could encourage online consumption of aging-friendly products among older populations.

Furthermore, this study found that economic cost has a significant positive influence on purchase intention, which indicates that when aging-friendly products are perceived as offering good value for money or being reasonably priced, older consumers are more likely to consider purchasing the products via online shopping. The findings are in line with the previous studies (9, 53, 83). This study also found that subjective norms have a significant positive influence on older adults’ purchase intention of aging-friendly products through online platforms. Which means that the family members, friends, caregivers, etc., who are important to older people are playing a crucial role in shaping the online purchasing behavior of older customers. The previous researchers also found consistent results (84).

Beyond the interpretation of individual findings, this study provides several theoretical contributions to literature on aging, technology adoption, and online consumption. First, the results broaden the applicability of the TAM by showing that emotional and socio-contextual conditions, such as digital literacy, trusting beliefs and technology anxiety also play crucial roles as traditional cognitive factors in influencing perceived usefulness and perceived ease of use among older adults. This study provides empirical support for this refinement. Second, the study deepens the understanding of aging-friendly consumption by focusing on online purchase intention within a product category that is directly related to older adults’ functional and daily living needs. By combining cognitive, emotional, and social determinants within a single analytical framework, this study presents a more comprehensive theoretical perspective on how older adults assess and adopt aging-friendly products in digital environments.

Although this study focuses on urban older adults in Guangzhou, several broader contextual factors in China warrant acknowledgement. Variations in smartphone access and digital infrastructure between rural and urban regions may influence older adults’ opportunities to engage in online shopping. In addition, changes in family structure and the reduced influence of traditional intergenerational support may shape the extent to which adult children or caregivers assist older adults in digital decision-making. Government-led digital inclusion initiatives designed to improve older adults’ digital participation may also gradually affect levels of digital literacy and online consumption nationwide. Recognizing these factors helps clarify the contextual boundaries of the study and provides a more accurate indication of the conditions under which the findings can be generalized.

## Theoretical implications

This study is based on the TAM and TPB theoretical models to investigate and understand older people’s online shopping behavior with aging-friendly products, mainly focusing on Guangzhou’s senior citizens over 60 years old, China. There are several theoretical gaps that this study could fill. First, this study revealed that perceived ease of use does not significantly influence purchase intention; the finding challenges the core of TAM. A large number of previous studies have shown that perceived ease of use is one of the main factors that affect consumers’ behavior (85–87). The main reason for the different results of this study may be that Guangzhou is a modern city with strong technological infrastructure and information popularization. With the improvement of basic digital literacy, the older people in Guangzhou may place greater priority on evaluations related to the final use results, such as usefulness, trust, and economic cost. Older adults’ consumption behavior in the digital environment might need a refining model to explain. This complements and extends previous findings. Second, this research found that trust beliefs in online shopping, economic cost, and subjective norms are significantly associated with purchase intention, which provided a broader range of influential constructs beyond the traditional TAM framework. Particularly, senior people’s emotional trust, value sensitivity, social approval, and acceptance are more salient in determining behavioral intentions in online shopping. The findings suggest the need for an integrated theoretical approach that incorporates social affective and cost-related variables into the TAM to address aging consumption in digital environments. Lastly, this study addressed an important research gap that in the intersection of digital aging, online shopping services, senior consumers behavior. Previous studies mostly focused on general technology adoption (88, 89) or health technology use (80). While this study specifically targeted online shopping for aging-friendly products, which is an emerging but underexplored aging consumption field.

### Practical impactions

This study can also provide some practical implications from different stakeholders’ perspectives. Such as older people, their family members, caregivers, online shopping platforms, aging-friendly products producers, and public agencies. First, older adults are encouraged to actively embrace new technologies and enhance their digital literacy, particularly in foundational skills such as online searching, product and price comparison, and secure online payment. Strengthening these competencies can enable older individuals to make better use of digital platforms, thereby improving their daily convenience and overall quality of life. Second, this study found that subjective norms are one of the major factors influencing older people’s purchase intentions. Therefore, family members or caregivers can play a proactive role in facilitating online shopping and provide both technical support and emotional encouragement to older adults. Co-shopping or caregiver-assisted tools interfaces could be developed to allow older people and family members/caregivers to collaborate to make online shopping and purchase management. Selecting the most fitting aging-friendly products not only can help older people themselves but also release some pressure of taking care of the older adults from the family members or caregivers. Third, from the online shopping platforms’ perspective, trustworthiness is the primary factor that determines the older adults’ attitude and behavior toward online shopping. Hence, online shopping platforms have to prioritize the design of trustworthy interfaces, such as implementing simplified user flows, senior-friendly shopping models with larger text, voice navigation, clear return policies, and users’ data and payment information security. Fourth, for the aging-friendly product manufacturers, clear communication of the practical function and benefits of the products is important. Product descriptions should emphasize how the particular aging-friendly products can enhance mobility, comfort, safety, or independence. Using visual demonstrations, testimonials from long-term users, and physicians’ recommendations can strengthen products’ competitive advantage. Due to the price sensitivity of older people, employing the tiered package price strategy, loyal discounts, and targeted promotions for senior users is recommended; those strategies could help to enhance the perceived value. Last, the policymakers in aging urban cities like Guangzhou should be encouraged to establish senior schools/universities to provide digital technology education to more older people, and government-supported education campaigns should raise awareness of digital fraud, which leads to technology anxiety. Educate them to learn how to be safe when shopping in the digital environment. Regulating platform accountability, particularly certification schemes for aging-friendly products and vendors, could further increase confidence in the online shopping environment.

### Limitations and further study

This study also has several limitations that should be considered in future research. First, the research was conducted exclusively in Guangzhou, China. Therefore, the findings may not be fully generalizable to other cities or countries with differing levels of digital infrastructure, socioeconomic conditions, or cultural contexts. Regional variations may influence older adults’ online shopping behaviors and attitudes. In addition, the study was conducted within an urban context, and broader structural factors in China may create variation that was not captured in the current sample. Differences in digital infrastructure across regions, unequal access to smartphones, and changes in intergenerational involvement may all influence how older adults engage with online shopping. Although these contextual elements fall outside the scope of the present analysis, they represent meaningful considerations for interpreting the findings. Future studies may extend this work by comparing urban and rural settings or by examining how evolving patterns of family support interact with digital adoption among older adults. Second, the study relied solely on a self-administered questionnaire to examine the variables influencing purchase intention. While this approach allows for efficient data collection, it may not fully capture the deeper perspectives of older adults. Future research could incorporate qualitative methods, such as in-depth interviews or focus groups, to explore older adults’ attitudes, concerns, and motivations in greater detail and provide richer insights into the underlying decision-making processes. Third, the senior participants in this study ranged in age from 60 to over 85 years. Their physical, cognitive, and emotional conditions may vary significantly within this age span, which potentially influences technology adoption behaviors. Future research could consider segmenting participants into two different age groups, such as younger-old and older-old groups, and testing the two groups as a moderating variable to examine potential differences in online shopping behavior, attitudes, and decision-making. Finally, where possible, the demographic characteristics of the sample were compared with official statistics released by the Guangzhou Municipal Bureau of Statistics. The gender composition and overall age structure of the sample were broadly consistent with those of the city’s older adult population. However, detailed demographic indicators such as education level, income distribution, digital literacy, and living arrangements with adult children are not publicly reported at the municipal level. As a result, a full variable-by-variable comparison with the population of Guangzhou could not be conducted. This limitation should be taken into account when interpreting the representativeness of the sample, and future studies may incorporate stratified sampling or link survey data with population-level datasets where available.

## Data Availability

The original contributions presented in the study are included in the article/Supplementary material, further inquiries can be directed to the corresponding author.

## References

[1] WHO (2024) Ageing and health world and health organization. Available online at: https://www.who.int/news-room/fact-sheets/detail/ageing-and-health?utm (Accessed February 4, 2025).

[2] WHO (2025). Aging and health in China. Available online at: https://www.who.int/china/health-topics/ageing?utm (Accessed February 4, 2025).

[3] Wang H Qin D Fang L Liu H Song P. Addressing healthy aging in China: practices and prospects. Biosci Trends. (2024) 18:212–8. doi: 10.5582/bst.2024.01180, 38987161

[4] Su S-W Wang D. Health-related quality of life and related factors among elderly persons under different aged care models in Guangzhou, China: a cross-sectional study. Qual Life Res. (2019) 28:1293–303. doi: 10.1007/s11136-019-02107-x, 30649697 PMC6470275

[5] Xi J-Y Liang B-H Zhang W-J Yan B Dong H Chen Y-Y . Effects of population aging on quality of life and disease burden: a population-based study. Glob Health Res Policy. (2025) 10:2. doi: 10.1186/s41256-024-00393-8, 39810282 PMC11731452

[6] Ying B Yao R. Self-perceived age and attitudes toward marketing of older consumers in China. J Fam Econ Iss. (2010) 31:318–27. doi: 10.1007/s10834-010-9199-y, 20835378 PMC2924498

[7] Roy G Basu R Ray S. Antecedents of online purchase intention among ageing consumers. Glob Bus Rev. (2023) 24:1041–57. doi: 10.1177/0972150920922010

[8] Wu J. Song S. 2021 Older adults’ online shopping continuance intentions: applying the technology acceptance model and the theory of planned behavior Int J Hum Comput Interact 37 10 938–948 doi: 10.1080/10447318.2020.1861419

[9] Yap Y-Y Tan S-H Tan S-K Choon S-W. Online grocery shopping intention: elderly's perspective in Malaysia. Heliyon. (2023) 9:e20827. doi: 10.1016/j.heliyon.2023.e20827, 37916123 PMC10616124

[10] Soh PY Heng HB Selvachandran G Anh LQ Chau HTM Son LH . Perception, acceptance and willingness of older adults in Malaysia towards online shopping: a study using the UTAUT and IRT models. J Ambient Intell Humaniz Comput. (2020):1–13. doi: 10.1007/s12652-020-01718-433224306

[11] Chakraborty R Lee J Bagchi-Sen S Upadhyaya S Rao HR. Online shopping intention in the context of data breach in online retail stores: an examination of older and younger adults. Decis Support Syst. (2016) 83:47–56. doi: 10.1016/j.dss.2015.12.007

[12] Kwon WS Noh M. The influence of prior experience and age on mature consumers' perceptions and intentions of internet apparel shopping. J Fashion Mark Manag: Int J. (2010) 14:335–49. doi: 10.1108/13612021011061825, 35579975

[13] Guido G Ugolini MM Sestino A. Active ageing of elderly consumers: insights and opportunities for future business strategies. SN Bus Econ. (2022) 2:8. doi: 10.1007/s43546-021-00180-4, 35018351 PMC8739688

[14] Charenkova J. “Parenting my parents”: perspectives of adult children on assuming and remaining in the caregiver's role. Front Public Health. (2023) 11:59–71. doi: 10.3389/fpubh.2023.1059006, 36875393 PMC9982148

[15] Zheng Z Ba M HS Omar Zaki H Tan QL. Impact of aging on consumer behavior: a review and research agenda. Int J Consum Stud. (2024) 48:46–59. doi: 10.1111/ijcs.70000

[16] Bardach SH Rhodus EK Parsons K Gibson AK. Older adults’ adaptations to the call for social distancing and use of technology: insights from socioemotional selectivity theory and lived experiences. J Appl Gerontol. (2021) 40:814–7. doi: 10.1177/0733464821996864, 33648357 PMC8238791

[17] Carstensen LL Hershfield HE. Beyond stereotypes: using socioemotional selectivity theory to improve messaging to older adults. Curr Dir Psychol Sci. (2021) 30:327–34. doi: 10.1177/09637214211011468, 34366582 PMC8340497

[18] Mei Y. Exploring the mechanisms driving elderly Fintech engagement: the role of social influence and the elderly’s digital literacy. Front Psychol. (2024) 15:1420147. doi: 10.3389/fpsyg.2024.1420147, 38974106 PMC11224538

[19] Schroeder T Dodds L Georgiou A Gewald H Siette J. Older adults and new technology: mapping review of the factors associated with older adults’ intention to adopt digital technologies. JMIR Aging. (2023) 6:e44564. doi: 10.2196/44564, 37191976 PMC10230357

[20] Nakayama J Morimoto K. Psychological elements for consideration in developing elderly-friendly products. Int Assoc Soc Design Res. (2007) 12:1–10.

[21] Li Z Xiang Y. Research on the development trends of user experience of age-appropriate products int and past decade. Adv Psychol. (2025) 15:232. doi: 10.12677/ap.2025.151029

[22] Cheng Y Xu X. Research on aging-friendly product design system under the perspective of elderly population. J Anhui Univ Technol. (2023) 40:48–52. doi: 10.3969/j.issn.1671-9247.2023.01.011

[23] Cheng Y Xu X Li B Huang Y. Design attributes and strategies of elderly-oriented products. J Fujian Univ Technol. (2022) 1:89–95.

[24] Gilster P. Digital literacy. Wiley Comput Pub. (1997) 25:1–16.

[25] Pangrazio L Godhe A-L Ledesma AGL. What is digital literacy? A comparative review of publications across three language contexts. E Learn Digit Media. (2020) 17:442–59. doi: 10.1177/2042753020946291

[26] Berkowsky RW Czaja SJ. Challenges associated with online health information seeking among older adults. In: Pak R Collins- Mclaughlin A editors. Aging, technology and health. Massachusetts (US): Elsevier (2018). 31–48.

[27] Mohammadyari S Singh H. Understanding the effect of e-learning on individual performance: the role of digital literacy. Comput Educ. (2015) 82:11–25. doi: 10.1016/j.compedu.2014.10.025

[28] Beldad A De Jong M Steehouder M. How shall i trust the faceless and the intangible? A literature review on the antecedents of online trust. Comput Hum Behav. (2010) 26:857–69. doi: 10.1016/j.chb.2010.03.013

[29] Chetioui Y Lebdaoui H Chetioui H. Factors influencing consumer attitudes toward online shopping: the mediating effect of trust. EuroMed J Bus. (2021) 16:544–63. doi: 10.1108/EMJB-05-2020-0046

[30] Javadi MHM Dolatabadi HR Nourbakhsh M Poursaeedi A Asadollahi AR. An analysis of factors affecting on online shopping behavior of consumers. Int J Mark Stud. (2012) 4:81. doi: 10.5539/ijms.v4n5p81

[31] Grimes GA Hough MG Mazur E Signorella ML. Older adults' knowledge of internet hazards. Educ Gerontol. (2010) 36:173–92. doi: 10.1080/03601270903183065

[32] Roalf DR Mitchell SH Harbaugh WT Janowsky JS. Risk, reward, and economic decision making in aging. J Gerontol B Psychol Sci Soc Sci. (2012) 67:289–98. doi: 10.1093/geronb/gbr099, 21926401 PMC3325085

[33] Wang T-L Tseng YF. A study of the effect on trust and attitude with online shopping. Int J Digit Soc. (2011) 2:433–40. doi: 10.1108/EMJB-05-2020-0046

[34] Cao C Huang S. How can SMEs boost trust through third-party means? Tracing the multi-dimensional institutional basis of online trust. IEEE Access. (2022) 10:127149–67. doi: 10.1109/ACCESS.2022.3226889

[35] Zhao J Wang H Zhang Y Huang Y. Trust in sharing accommodation sector: an institution-based trust perspective. Internet Res. (2023) 33:1399–421. doi: 10.1108/INTR-04-2021-0261

[36] Fruchter GE Reutterer T Dickert S Vacondio M. Dynamic formation of quality expectations: theory and empirical evidence. Rev Mark Sci. (2023) 21:35–75. doi: 10.1515/roms-2022-0096

[37] Bulsara HP Vaghela PS. Trust and online purchase intention: a systematic literature review through meta-analysis. Int J Electron Bus. (2023) 18:148–64. doi: 10.1504/IJEB.2023.130155

[38] Simonson MR Maurer M Montag-Torardi M Whitaker M. Development of a standardized test of computer literacy and a computer anxiety index. J Educ Comput Res. (1987) 3:231–47. doi: 10.2190/7CHY-5CM0-4D00-6JCG

[39] Vroman KG Arthanat S Lysack C. Who over 65 is online? Comput Hum Behav. (2015) 43:156–66. doi: 10.1016/j.chb.2014.10.018

[40] Flick C Zamani ED Stahl BC Brem A. The future of ICT for health and ageing: unveiling ethical and social issues through horizon scanning foresight. Technol Forecast Soc Change. (2020) 155:119995. doi: 10.1016/j.techfore.2020.119995

[41] Meuter ML Ostrom AL Bitner MJ Roundtree R. The influence of technology anxiety on consumer use and experiences with self-service technologies. J Bus Res. (2003) 56:899–906. doi: 10.1016/S0148-2963(01)00276-4

[42] Chen K Chan AH. Predictors of gerontechnology acceptance by older Hong Kong Chinese. Technovation. (2014) 34:126–35. doi: 10.1016/j.technovation.2013.09.010

[43] Tsai J-M Cheng M-J Tsai H-H Hung S-W Chen Y-L. Acceptance and resistance of telehealth: the perspective of dual-factor concepts in technology adoption. Int J Inf Manag. (2019) 49:34–44. doi: 10.1016/j.ijinfomgt.2019.03.003

[44] An J Zhu X Wan K Xiang Z Shi Z An J . Older adults’ self-perception, technology anxiety, and intention to use digital public services. BMC Public Health. (2024) 24:3533–53. doi: 10.1186/s12889-024-21088-2, 39702092 PMC11657182

[45] Kim H-N Freddolino PP Greenhow C. Older adults’ technology anxiety as a barrier to digital inclusion: a scoping review. Educ Gerontol. (2023) 49:1021–38. doi: 10.1080/03601277.2023.2202080

[46] Huang T. Using SOR framework to explore the driving factors of older adults smartphone use behavior. Hum Soc Sci Commun. (2023) 10:1–16. doi: 10.1057/s41599-023-02221-9, 39310270

[47] Davis FD. Perceived usefulness, perceived ease of use, and user acceptance of information technology. MIS Q. (1989) 13:319–40. doi: 10.2307/249008, 41264861

[48] Ahn T Ryu S Han I. The impact of the online and offline features on the user acceptance of internet shopping malls. Electron Commer Res Appl. (2004) 3:405–20. doi: 10.1016/j.elerap.2004.05.001

[49] Lee M-C. Factors influencing the adoption of internet banking: an integration of TAM and TPB with perceived risk and perceived benefit. Electron Commer Res Appl. (2009) 8:130–41. doi: 10.1016/j.elerap.2008.11.006

[50] Erjavec J Manfreda A. Online shopping adoption during COVID-19 and social isolation: extending the UTAUT model with herd behavior. J Retail Consum Serv. (2022) 65:102867. doi: 10.1016/j.jretconser.2021.102867

[51] Ruangkana V. Kessuvan A. 2019. Factors affecting the elderly's adoption of online purchasing. Science, and industrial applications, 1–5. 2019 International Conference on Engineering, Science, and Industrial Applications (ICESI)

[52] Ramirez-Correa P Grandon E Ramirez-Santana M Arenas-Gaita J Rondan-Cataluna F. Explaining the consumption technology acceptance in the elderly post-pandemic: effort expectancy does not matter. Behav Sci. (2023) 13:87–102. doi: 10.3390/bs13020087 36829316 PMC9952286

[53] Li S Huang J Lu C Wu Z Antwi-Afari MF. Investigating the influencing factors of the purchase intention of the continuing care retirement community: a case study of Shenzhen. Sustainability. (2024) 16:22–39. doi: 10.3390/su16052201

[54] Zhou C Qian Y Kaner J. A study on smart home use intention of elderly consumers based on technology acceptance models. PLoS One. (2024) 19:e0300574. doi: 10.1371/journal.pone.0300574, 38536849 PMC10971693

[55] Kim J-J. The effects of elderly (senior) buying factors and satisfaction on retailer's online shopping. J Distrib Sci. (2017) 15:43–52. doi: 10.15722/jds.15.7.201707.43

[56] Berg H Liljedal KT. Elderly consumers in marketing research: a systematic literature review and directions for future research. Int J Consum Stud. (2022) 46:1640–64. doi: 10.1111/ijcs.12830

[57] Peng X Chen Q. A brief discussion on China's silver economy. Chin Soc Secur Rev. (2022) 6:49–66.

[58] Beck L Ajzen I. Predicting dishonest actions using the theory of planned behavior. J Res Pers. (1991) 25:285–301. doi: 10.1016/0092-6566(91)90021-H

[59] Jacelon CS. Theoretical perspectives of perceived control in older adults: a selective review of the literature. J Adv Nurs. (2007) 59:1–10. doi: 10.1111/j.1365-2648.2007.04320.x, 17543008

[60] Courneya K. S. Nigg C. R. Estabrooks P. A. 2013 Relationships among the theory of planned behaviour, stages of change, and exercise behaviour in older persons over a three year period. In: Abraham C Norman P Conner M editors. Understanding and changing health behaviour. London, UK: Psychology Press. 189–205. Available online at: https://www.taylorfrancis.com/chapters/edit/10.4324/9781315080055-13/relationships-among-theory-planned-behaviour-stages-change-exercise-behaviour-older-persons-three-year-period-kerry-courneya-claudio-nigg-paul-estabrooks (Accessed February 26, 2025).

[61] Peña-García N Gil-Saura I Rodríguez-Orejuela A Siqueira-Junior JR. Purchase intention and purchase behavior online: a cross-cultural approach. Heliyon. (2020) 6:e04284–11. doi: 10.1016/j.heliyon.2020.e04284 32613132 PMC7322128

[62] Bamberg S Hunecke M Blöbaum A. Social context, personal norms and the use of public transportation: two field studies. J Environ Psychol. (2007) 27:190–203. doi: 10.1016/j.jenvp.2007.04.001

[63] Irawan RL Hurriyati R. The effects of subjective norm on the intention to use of the online shopping customers in Bandung. Manag Entrepreneurship. (2021):367–70. doi: 10.2991/aebmr.k.210831.072

[64] Tu J Shen M Zhong J Yuan G Chen M. The perceptions and experiences of mobile health technology by older people in Guangzhou, China: a qualitative study. Front Public Health. (2021) 9:683712. doi: 10.3389/fpubh.2021.683712, 34249848 PMC8267812

[65] Chen H Ge J He W. Quantifying urban vitality in Guangzhou through multi-source data: a comprehensive analysis of land use change, streetscape elements, POI distribution, and smartphone-GPS data. Land. (2025) 14:1309. doi: 10.3390/land14061309

[66] Cui K Zou W Ji X Zhang X. Does digital technology make people healthier: the impact of digital use on the lifestyle of Chinese older adults. BMC Geriatr. (2024) 24:85. doi: 10.1186/s12877-023-04651-1, 38254001 PMC10804579

[67] Zhou XF Chen L. Digital health care in China and access for older people. Lancet Public Health. (2021) 6:e873–4. doi: 10.1016/S2468-2667(21)00051-7, 34197810

[68] ChinaYearbooks. (2023). Guangzhou statistical yearbook 2022. Available online at: https://www.chinayearbooks.com/guangzhou-statistical-yearbook-2022.html (Accessed October 12, 2025).

[69] NBS (2025). National Bureau of Statistics of China. Available online at: https://www.stats.gov.cn/english/StatisticalCommuniqu/ (Accessed October 12, 2025).

[70] Hair JF Risher JJ Sarstedt M Ringle CM. When to use and how to report the results of PLS-SEM. Eur Bus Rev. (2019) 31:2–24. doi: 10.1108/EBR-11-2018-0203

[71] Cheung GW Cooper-Thomas HD Lau RS Wang LC. Reporting reliability, convergent and discriminant validity with structural equation modeling: a review and best-practice recommendations. Asia Pac J Manag. (2024) 41:745–83. doi: 10.1007/s10490-023-09880-x

[72] Hair J Hollingsworth CL Randolph AB Chong AYL. An updated and expanded assessment of PLS-SEM in information systems research. Ind Manag Data Syst. (2017) 117:442–58. doi: 10.1108/IMDS-04-2016-0130

[73] Henseler J Ringle CM Sarstedt M. A new criterion for assessing discriminant validity in variance-based structural equation modeling. J Acad Mark Sci. (2015) 43:115–35. doi: 10.1007/s11747-014-0403-8

[74] Schober P Boer C Schwarte LA. Correlation coefficients: appropriate use and interpretation. Anesth Analg. (2018) 126:1763–8. doi: 10.1213/ANE.0000000000002864, 29481436

[75] Adeboye N Fagoyinbo I Olatayo T. Estimation of the effect of multicollinearity on the standard error for regression coefficients. J Undergrad Math. (2014) 10:16–20. doi: 10.9790/5728-10411620

[76] Kock N Lynn GS. Lateral collinearity and misleading results in variance-based SEM: an illustration and recommendations. J Assoc Inf Syst. (2012) 13:2. doi: 10.17705/1jais.00302

[77] Henseler J Hubona G Ray PA. Using PLS path modeling in new technology research: updated guidelines. Ind Manag Data Syst. (2016) 116:2–20. doi: 10.1108/IMDS-09-2015-0382

[78] Wetzels M Odekerken-Schröder G Van Oppen C. Using PLS path modeling for assessing hierarchical construct models: guidelines and empirical illustration. MIS Q. (2009) 33:177–95. doi: 10.2307/20650284, 39964225

[79] Zhang L Anjum MA Wang Y. The impact of trust-building mechanisms on purchase intention towards metaverse shopping: the moderating role of age. Int J Hum Comput Interact. (2024) 40:3185–203. doi: 10.1080/10447318.2023.2184594

[80] Wang Y Lu L Zhang R Ma Y Zhao S Liang C. The willingness to continue using wearable devices among the elderly: SEM and FsQCA analysis. BMC Med Inform Decis Mak. (2023) 23:218. doi: 10.1186/s12911-023-02336-8, 37845659 PMC10577990

[81] Li J Ma Q Chan AH Man S. Health monitoring through wearable technologies for older adults: smart wearables acceptance model. Appl Ergon. (2019) 75:162–9. doi: 10.1016/j.apergo.2018.10.006, 30509522

[82] Xu Y Wang Y Khan A Zhao R. Consumer flow experience of senior citizens in using social media for online shopping. Front Psychol. (2021) 12:732104. doi: 10.3389/fpsyg.2021.732104, 34603153 PMC8480428

[83] Afendi A. The effect of price sensitivity on purchase intention is mediated by advocacy and emotion on consumers of pharmaceutical companies in Jakarta. J Soc Res. (2023) 2:1737–52. doi: 10.55324/josr.v2i5.881

[84] Lim-Ai L Cynthia P Cynthia P. Influence of subjective norms on shaping consumer purchase intentions: a structured review. Malays J Bus Econ. (2024) 11:35–52. doi: 10.51200/mjbe.v11i2.5782

[85] Hansen JM Saridakis G Benson V. Risk, trust, and the interaction of perceived ease of use and behavioral control in predicting consumers’ use of social media for transactions. Comput Hum Behav. (2018) 80:197–206. doi: 10.1016/j.chb.2017.11.010

[86] Hyun H Thavisay T Lee SH. Enhancing the role of flow experience in social media usage and its impact on shopping. J Retail Consum Serv. (2022) 65:102492. doi: 10.1016/j.jretconser.2021.102492

[87] Wilson N Keni K Tan PHP. The role of perceived usefulness and perceived ease-of-use toward satisfaction and trust which influence computer consumers’ loyalty in China. Gadjah Mada Int J Bus. (2021) 23:262–94. doi: 10.3316/INFORMIT.147511565887487

[88] Copeland LR Bhaduri G Huang O. Understanding Chinese gen Z and their online shopping intentions through TAM. Asia Pac J Mark Logist. (2023) 35:2361–76. doi: 10.1108/APJML-03-2022-0241

[89] Yadegari M Mohammadi S Masoumi AH. Technology adoption: an analysis of the major models and theories. Technol Anal Strateg Manag. (2024) 36:1096–110. doi: 10.1080/09537325.2022.2071255

